# Injection of Quinine in Gonorrhœa

**Published:** 1876-08

**Authors:** 


Injection of Quinine in Gonorrhœa.—Radha Nauth Roy, Asst. Surg., extols (*Indian Med. Gazette*, May, 1876,) the efficacy of injections of quinia in gonorrhœa. He states: "I was once tempted to try it in a case of acute gonorrhœa, where scalding was unbearable, and discharge profuse, and to my utter surprise, after the third day I found the man quite relieved. He described to me the soothing effect of the injection as something cold like ice. The discharge was so much diminished that his clothes were scarcely stained after the third day. There was no more incessant desire to void the bladder, and he was to all appearance comfortable. My success in this case made me bold enough to use it in other cases, and I have invariably found the disease yield both in its acute and chronic stage under its influence. It acts as a tonic and astringent to the mucous membrane of the urethra. I have also used it in some cases of cystitis with much benefit. I generally use it dissolved in sulphuric acid dil. mixed with rose-water.—Two grains of quinine sulph. dissolved in acid. sulph. dil. *m*viij. or *m*x., and mixed with an ounce of rose-water—to be used twice for injection. At the same time I give copaiba mixture to my patients. In almost all the cases I have found it act like a charm. The disease is generally cured within a week, but chronic cases take a longer time. In a few acute cases it took more than a fortnight, but the delay in them was attributable to their irregular habits during the treatment.—*Am. Jour. Med. Sciences.*

